# Associations of Serum 25-Hydroxyvitamin D_3_ Levels with Visceral Adipose Tissue in Chinese Men with Normal Glucose Tolerance

**DOI:** 10.1371/journal.pone.0086773

**Published:** 2014-01-22

**Authors:** Yaping Hao, Xiaojing Ma, Yun Shen, Jie Ni, Yuqi Luo, Yunfeng Xiao, Yuqian Bao, Weiping Jia

**Affiliations:** 1 Department of Endocrinology and Metabolism, Shanghai Jiao Tong University Affiliated Sixth People’s Hospital, Shanghai Clinical Center for Diabetes, Shanghai Key Clinical Center for Metabolic Disease, Shanghai Diabetes Institute, Shanghai Key Laboratory of Diabetes Mellitus, Shanghai, China; 2 Department of Radiology, Shanghai Jiao Tong University Affiliated Sixth People’s Hospital, Shanghai, China; University of Tennessee, United States of America

## Abstract

**Objective:**

Decreased serum vitamin D level is a common observation in obese adults. Since no Chinese population-based study has yet evaluated the relationship between serum vitamin D levels and the accurate adiposity variables, this study investigated the association of serum vitamin D (assessed by 25-hydroxyvitamin D_3_ [25(OH)D_3_]) levels with precise body fat content and distribution in a cohort of Chinese men.

**Methods:**

Serum samples were collected from a total of 567 men with normal glucose tolerance (NGT) for assessment by electrochemiluminescence immunoassay to measure 25(OH)D_3_ levels. In addition, each participant underwent bioelectrical impedance analysis to quantify total body fat and magnetic resonance imaging to measure visceral fat area (VFA) and subcutaneous fat area (SFA).

**Results:**

Overweight/obese (BMI ≥25 kg/m^2^) subjects had significantly lower serum 25(OH)D_3_ levels than non-overweight/non-obese (BMI <25 kg/m^2^) subjects (*P*  = 0.029). Greater fat mass and VFA were accompanied by a downward trend in serum 25(OH)D_3_ levels (*P* for trend <0.01). Among overweight/obese subjects, those with body fat percent ≥25% also had significantly lower serum 25(OH)D_3_ levels (*P* <0.05). Moreover, participants with VFA ≥80 cm^2^ had significantly lower serum 25(OH)D_3_ (*P* <0.05), regardless of BMI value. VFA was independently correlated with serum 25(OH)D_3_ levels (*β*  =  −0.023, *P* <0.001), even after adjustments for confounding factors. In addition, serum 25(OH)D_3_ levels were found to decrease by 0.26 ng/mL per 10 cm^2^ increment of VFA.

**Conclusions:**

Serum 25(OH)D_3_ levels were inversely associated with VFA in Chinese men with NGT.

## Introduction

The primary biological role of vitamin D, a lipid-soluble vitamin, is the regulation of calcium and phosphorus metabolism; as such, proper levels of vitamin D are critical for establishment of bone health and its maintenance throughout life [1]. Recent studies have indicated that perturbed vitamin D status may also contribute to obesity, metabolic syndrome and cardiovascular disease [2].

In recent years, the relationship between Vitamin D and obesity has received extensive attention. Animal studies found that when mouse 3T3-L1 preadipocytes were exposed to vitamin D, the formation of adipocytes was suppressed as a result of inhibited proliferation and differentiation [3]. In addition, when high-dose vitamin D was delivered as a dietary supplement, along with high whey protein and calcium, male Wistar rats experienced a reduction in fat mass and an increase in lean mass [4].

As the main circulating form of vitamin D, serum 25-hydroxyvitamin D [25(OH)D], consisted of 25(OH)D_2_ and 25(OH)D_3_, is used as a clinical marker to evaluate vitamin D nutritional status. Additionally, more than 95% of 25(OH)D, measurable in serum, is 25(OH)D_3_ [5]. Consistent with the experimental studies, clinical evidence has indicated that serum 25(OH)D levels decrease significantly in obese subjects and has shown that this decrease is closely correlated with fat distribution [6], [7]. Still other studies have implicated the decreased level of serum 25(OH)D as a risk factor for obesity and its related metabolic disorders [8].

Anthropometric indices for obesity and abdominal obesity, such as body mass index (BMI) and waist circumference (WC), have been used widely in the previous studies examining the relationship between obesity and vitamin D. Additionally, the differences in serum 25(OH)D levels associated with sex and race are well established [9]. However, the associations of serum vitamin D levels with accurate adiposity variables remain unknown within the Chinese population.

Therefore, in the present study, magnetic resonance imaging (MRI) was used to evaluate visceral fat area (VFA) and subcutaneous fat area (SFA) as accurate measurements for visceral obesity. Body composition (as a precise evaluation of total body fat content) was determined by bioelectrical impedance. The study was designed to evaluate the association between serum vitamin D levels (assessed by serum 25(OH)D_3_) and body fat as well as fat distribution in a cohort of Chinese men with normal glucose tolerance (NGT), in order to minimize the known influence of hyperglycemia, including impaired glucose regulation (IGR) and diabetes, on serum 25(OH)D_3_ levels [10].

## Subjects and Methods

### Study subjects

The study was conducted in accordance with the Declaration of Helsinki and approved by the Ethics Committee of Shanghai Jiao Tong University Affiliated Sixth People’s Hospital. All study participants provided written informed consent prior to enrollment.

The 1003 adult men with no previous history of diabetes who participated in the Shanghai Obesity Study (SHOS) were considered for study enrollment [11]. This overall population included 289 subjects from the Gonghexin community who participated during August to September in 2010 and 714 subjects from the Tianmuxi and Daning communities who participated during April to September in 2011. All of the participants underwent MRI scan to obtain abdominal fat area measurements and completed a standardized questionnaire to identify history of present and previous illness, medical therapy, physical activity, and smoking status.

This study participants was filtered according to the following exclusion criteria: newly diagnosed type 2 diabetes mellitus or IGR (n  =  266); liver and kidney dysfunction (n  =  45); hyperthyroidism or hypothyroidism (n  =  31); serum calcium levels ≥10.5 mg/dL (n  =  4) [12]; current corticosteroids therapy or supplemental calcium/vitamin D intake (n  =  4); psychiatric disease, severe disability or occurrence of bone fracture within the past six months (n  =  3); history of cardiovascular disease (n  =  27); recent infection (n  =  24); serum C reactive protein >10 mg/L (n  =  22); presence of tumor (n  =  5); severe anemia (n  =  5). Finally, 567 subjects with NGT were included in the analysis.

### Anthropometric measurements

BMI (kg/m^2^) was calculated based on the height (to the nearest 0.1 cm) and weight (to the nearest 0.1 kg). Waist circumference was measured starting at the midpoint of the inferior border of the lowest rib and following the iliac crest on the mid-axillary line by around the abdomen. Resting blood pressure was calculated as the average value of three measurements taken at 3 min intervals.

### Body fat measurements

Body composition, consisting of fat mass (FM) and free fat mass (FFM), was estimated by the BC-420 Tanita Body Composition Analyzer (Tanita Corp., Tokyo, Japan). Percentage of body fat (fat%) was calculated as FM (kg)/[FM (kg)+FFM (kg)]. Abdominal MRI scans were performed on the Archiva 3.0T Clinical MRI Scanner (Philips Medical System, Amsterdam, The Netherlands) at the level between the L4 and L5 vertebrae with the participant in the supine position [13]. VFA and SFA were calculated using the Slice-O-Matic Image Analysis Software (version 4.2; Tomovision Inc., Montreal, QC, Canada).

### Biochemical assessments

All subjects underwent a 75-g oral glucose tolerance test after 10-h overnight fasting, and blood samples were collected to measure 25(OH)D_3_ levels as well as other biochemical parameters. Fasting plasma glucose (FPG) and 2h postprandial glucose (2hPG) were measured by the glucose oxidase method. Lipid profiles, including total cholesterol (TC) and triglycerides (TG), were determined using the standard enzymatic methods, while low-density lipoprotein cholesterol (LDL-c) and high-density lipoprotein cholesterol (HDL-c) were determined by the direct assay method. All of the above measurements were carried out on a Hitachi 7600–120 auto-analyser (Tokyo, Japan). Serum fasting insulin (FINS) level was measured by electrochemiluminescence immunoassay, and the intra- and inter-assay coefficients of variation were 1.7% and 2.5%, respectively. Insulin resistance (IR) was estimated by the homeostasis model assessment index (HOMA-IR) [14]. Only serum 25(OH)D_3_ levels, but not the total 25(OH)D, was measured by electrochemiluminescence immunoassay (Roche Diagnostics GmbH, Mannheim, Germany), and the intra- and inter-assay coefficients of variation were 5.6% and 8.0%, respectively. The lower limit of 25(OH)D_3_ detection was <4 ng/mL.

### Definition

Levels of physical activity were classified as light, moderate or high according to the 2001 International Physical Activity Questionnaire (IPAQ) [15]. Hypertension was defined as systolic blood pressure (SBP) ≥140 mmHg and/or diastolic blood pressure (DBP) ≥90 mmHg, or current treatment for hypertension, according to the 1999 World Health Organization’s (WHO) Hypertension Guidelines [16]. Overweight/obesity was diagnosed when BMI was ≥25.0 kg/m^2^, according to the 1999 WHO criteria [17], and as fat% ≥ 25%, according to the WHO definition of obesity for men [18]. In addition, visceral obesity was defined as VFA ≥80 cm^2^. Dyslipidemia was defined as receipt of treatment of lipid abnormalities or lipid values over the boundary value according to the following criteria published by the Joint Committee for Developing Chinese Guidelines on Prevention and Treatment of Dyslipidemia in Adults [19]: hypercholesteremia: TC ≥5.18 mmol/L; hypertriglyceridemia: TG ≥1.70 mmol/L; high LDL-c: LDL-c ≥3.37 mmol/L; low HDL-c: HDL-c <1.04 mmol/L.

### Statistical analysis

All statistical analyses were performed by SPSS statistical software (version 16.0; SPSS Inc., Chicago, IL, USA). The one-sample Kolmogorov-Smirnov test was used to determine data normality; normally distributed data were expressed as mean±standard deviation (SD) and skewed data were expressed as median with interquartile range. For continuous variables, the unpaired Student’s *t*-test and the Mann-Whitney U-test were used for between-group comparisons of normally distributed and skewed data, respectively. For categorical variables, the χ^2^ test was used for between-group comparisons. Correlation coefficients between serum 25(OH)D_3_ levels and clinical parameters were determined by simple and partial correlation analyses, as appropriate. Multiple stepwise regression analysis was performed to identify independent associations of obesity-related variables and other parameters with serum 25(OH)D_3_ levels, after adjusting for potential confounders, while linear regression analysis was carried out to estimate the relationship between serum 25(OH)D_3_ levels and VFA. The threshold for statistical significance was a two-tailed *P*-value of <0.05.

## Results

### Clinical characteristics of study participants

The median level of age was 56.4 years old (interquartile range: 50.7–61.0). As shown in Table 1, there were no differences between overweight/obese (BMI ≥25 kg/m^2^) and non-overweight/non-obese (BMI <25 kg/m^2^) groups in age, 2hPG, TG or LDL-c levels, dyslipidemia (including hypercholesteremia, hypertriglyceridemia, high LDL-c and low HDL-c) frequency, physical activity, smoking status, or lipid-lowering therapy (all *P* >0.05). The serum 25(OH)D_3_ concentrations (overall range: 4.3–40.6 ng/mL) were lower in the overweight/obese individuals than in their non-overweight/non-obese counterparts. In addition, the overweight/obese individuals exhibited lower TC, HDL-c (both *P* <0.01) and higher BMI, WC, FM, FFM, fat%, VFA, SFA, SBP, DBP, FPG, FINS, HOMA-IR, and frequency of both hypertension as well as antihypertensive therapy (all *P* <0.05).

**Table 1:** Demographic and clinical characteristics of study participants.

Variables	Total	BMI<25 kg/m^2^	BMI≥25 kg/m^2^	*P*
N	567	404	163	-
Age (year)	56.4(50.7–61.0)	56.5(50.4–61.1)	55.9(51.3–60.5)	0.906
BMI (kg/m^2^)	23.8±2.8	22.4±1.8	27.2±2.0	<0.001
WC (cm)	84.9±8.6	81.5±6.5	93.3±7.1	<0.001
FM (kg)	15.1(11.6–18.2)	13.2(10.7–15.8)	20.1(17.0–24.3)	<0.001
FFM (kg)	53.6±5.6	51.7±4.4	58.5±5.5	<0.001
Fat% (%)	21.9±5.1	20.2±4.1	26.2±4.6	<0.001
VFA (cm^2^)	91.4±38.7	79.6±33.5	120.7±35.3	<0.001
SFA (cm^2^)	138.3(110.0–175.6)	122.7(101.2–148.9)	190.0(157.7–223.2)	<0.001
SBP (mmHg)	122.0(114.7–131.7)	120.7(112.4–130.0)	129.3(120.0–140.0)	<0.001
DBP (mmHg)	80.0(72.7–84.7)	79.3(71.3–82.0)	80.7(77.3–88.7)	<0.001
FPG (mmol/L)	5.2±0.4	5.2±0.4	5.3±0.4	<0.001
2hPG (mmol/L)	5.8±1.1	5.8±1.1	5.9±1.1	0.526
TC (mmol/L)	5.1±0.9	5.1±0.9	4.9±0.8	0.006
TG (mmol/L)	1.4(1.0–1.9)	1.4(1.0–1.9)	1.5(1.1–2.1)	0.052
HDL-c (mmol/L)	1.3(1.1–1.5)	1.3(1.1–1.5)	1.1(1.0–1.3)	<0.001
LDL-c (mmol/L)	3.2±0.8	3.2±0.8	3.1±0.7	0.288
FINS (mU/L)	6.5(4.5–9.2)	5.7(4.0–7.6)	8.9(6.4–12.6)	<0.001
HOMA-IR	1.5(1.0–2.2)	1.3(0.9–1.9)	2.1(1.5–3.1)	<0.001
25(OH)D_3_ (ng/mL)	15.5±5.6	15.8±5.7	14.7±5.3	0.029
Hypercholesteremia, N(%)	234(41.3)	173(42.8)	61(37.4)	0.259
Hypertriglyceridemia, N(%)	195(34.4)	134(33.2)	61(37.4)	0.379
High LDL-c, N(%)	209(36.9)	153(37.9)	56(34.4)	0.444
Low HDL-c, N(%)	109(19.2)	71(17.6)	38(23.3)	0.126
Hypertension, N(%)	187(33.0)	109(27.0)	78(47.9)	<0.001
Physical activity (high), N(%)	219(38.6)	153(37.9)	66(40.5)	0.844
Current smoking, N(%)	322(56.8)	235(58.2)	87(53.4)	0.557
Lipid-lowering therapy, N(%)	6(1.1)	3(0.7)	3(1.8)	0.361
Anti-hypertensives, N(%)	85(15.0)	51(12.6)	34(20.9)	0.019

Abbreviation: BMI, body mass index; WC, waist circumference; FM, fat mass; FFM, free fat mass; Fat%, percentage of body fat; VFA, visceral fat area; SFA, subcutaneous fat area; SBP, systolic blood pressure; DBP, diastolic blood pressure; FPG, fasting plasma glucose; 2hPG, 2-h postprandial plasma glucose; TC, total cholesterol; TG, triglyceride; HDL-c, high-density lipoprotein cholesterol; LDL-c, low-density lipoprotein cholesterol; FINS, fasting insulin; HOMA-IR, homeostasis model assessment for insulin resistance; 25(OH)D_3_, 25-hydroxyvitamin D_3_. Data were expressed as mean ±SD for normal distribution variables or median (interquartile range) for skewed distribution variables.

### Association of serum 25(OH)D_3_ levels with anthropometric and biochemical parameters

Serum 25(OH)D_3_ levels showed a significantly inverse correlation with obesity-related parameters (BMI, WC, FM, fat%, VFA and SFA) and TG (*P* <0.05). However, after adjustment for age and BMI, only FM, fat%, VFA and TG retained the significant inverse correlation with serum 25(OH)D_3_ levels (all *P* <0.01) (Table 2).

**Table 2:** Correlation of 25(OH)D_3_ with anthropometric parameters and biochemical indexes.

variables	25(OH)D_3_		25(OH)D_3_ (adjust for age and BMI)	
	*r*	*P*	*R*	*P*
Age	0.002	0.968	-	-
BMI	–0.101	0.016	-	-
WC	–0.115	0.006	–0.055	0.192
FM	–0.132	0.002	–0.156	<0.001
FFM	–0.044	0.291	0.036	0.398
Fat%	–0.165	<0.001	–0.137	0.001
VFA	–0.182	<0.001	–0.154	<0.001
SFA	–0.112	0.008	–0.061	0.148
SBP	0.010	0.820	0.007	0.872
DBP	–0.007	0.861	–0.006	0.882
FPG	0.038	0.367	0.062	0.143
2hPG	–0.010	0.820	–0.003	0.938
TC	–0.009	0.837	–0.017	0.679
TG	–0.108	0.010	–0.113	0.007
HDL-c	0.033	0.438	0.016	0.713
LDL-c	–0.013	0.762	–0.011	0.801
FINS	–0.063	0.134	–0.033	0.430
HOMA-IR	–0.053	0.209	–0.025	0.558
Physical activity	0.065	0.122	0.069	0.103
Current smoking	0.015	0.718	0.016	0.702

Abbreviation: 25(OH)D_3_, 25-hydroxyvitamin D_3_; BMI, body mass index; DBP, diastolic blood pressure; Fat%, percentage of body fat; FFM, free fat mass; FINS, fasting insulin; FPG, fasting plasma glucose; FM, fat mass; HDL-c, high-density lipoprotein cholesterol; HOMA-IR, homeostasis model assessment for insulin resistance; 2hPG, 2h postprandial plasma glucose; LDL-c, low-density lipoprotein cholesterol; SBP, systolic blood pressure; SFA, subcutaneous fat area; TC, total cholesterol; TG, triglyceride; VFA, visceral fat area; WC, waist circumference.

### Relationship between 25(OH)D_3_ and total body fat and VFA

**Figure 1:**
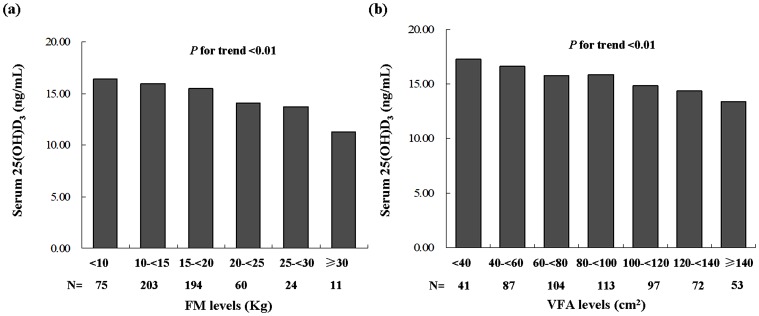
Serum 25(OH)D_3_ with FM (a) or VFA (b) levels. (a) Subjects were stratified into 6 subgroups according to FM levels (5 kg increments). (b) Subjects were stratified into 7 subgroups according to VFA levels (20 cm^2^ increments).

When the overall subjects were stratified by 5 kg increments of FM, the serum 25(OH)D_3_ levels were found to decrease in accordance with the increment, as expected (*P* for trend <0.01) (Fig. 1a). Moreover, when the groups were stratified by 20 cm^2^ increments of VFA, the same trend of descending serum 25(OH)D_3_ levels was observed (*P* for trend <0.01) (Fig. 1b).

**Figure 2:**
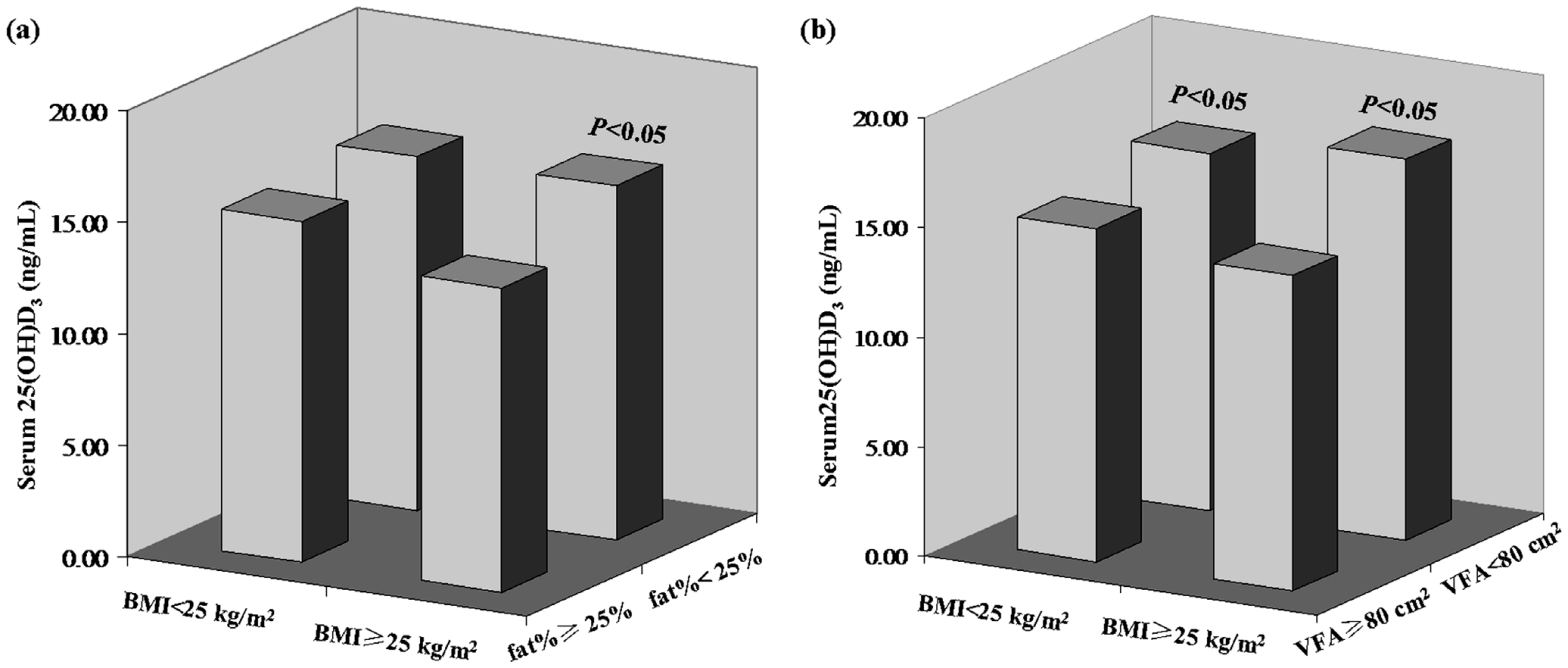
Serum 25(OH)D_3_ according to different fat% (a) / VFA (b) levels within similar BMI categories. (a) In the same BMI category, subjects were stratified into fat% ≥ 25% and fat% < 25% subgroups. (b) In the same BMI category, subjects were stratified into VFA ≥ 80 cm^2^ and VFA < 80 cm^2^ subgroups.

When the overweight/obese and non-overweight/non-obese groups were stratified by fat% (<25% vs. ≥25%), among the overweight/obese individuals, those with fat% ≥ 25% showed significantly lower serum 25(OH)D_3_ levels compared to those with fat% < 25% (*P* <0.05) (Fig. 2a). The non-overweight/non-obese individuals showed no significant difference in serum 25(OH)D_3_ levels between the fat% stratified subgroups.

Finally, when the overweight/obese and non-overweight/non-obese groups were further stratified according to the cutoff of visceral obesity, it was found that within the same BMI categories, serum 25(OH)D_3_ levels were significantly lower for those individuals with VFA ≥80 cm^2^, compared to those with VFA <80 cm^2^ (*P* < 0.05) (Fig. 2b).

### Variables independently associated with serum 25(OH)D_3_ levels

In order to assess the variables independently associated with serum 25(OH)D_3_ levels, multiple stepwise regression analysis identified the following variables as independent variables: obesity-related parameters (including BMI, WC, FM, FFM, fat%, VFA, and SFA), age, SBP, DBP, FPG, 2hPG, TC, TG, LDL-c, HDL-c, FINS, physical activity, smoking status, and receipt of lipid-lowing therapy or antihypertensive therapy. Three regression models were established according to the selection of different obesity-related variables. In model 1, the anthropometric variables (BMI and WC) were applied. We found that WC (*β*  =  –0.065, *P*  =  0.017) and TG (*β*  =  –0.524, *P*  =  0.009) showed independent associations with serum 25(OH)D_3_. Both model 2 (using accurate adiposity parameters including FM, FFM, fat%, VFA and SFA) and the expanded model 3 (using the additional anthropometric variables including BMI and WC) showed independent associations of serum 25(OH)D_3_ with VFA (*β*  =  –0.023, *P* <0.001) and TG (*β*  =  –0.415, *P*  =  0.041).

In addition, linear regression analysis, with serum 25(OH)D_3_ levels set as the dependent variable and VFA set as the independent variable, estimated serum 25(OH)D_3_ levels to be decreased by 0.26 ng/mL for each 10 cm^2^ incremental change in VFA.

## Discussion

To our knowledge, the study described herein has provided the first evidence of an association of serum 25(OH)D_3_ levels with precise body fat content and distribution in Chinese men with normal glucose tolerance. In particular, the data demonstrated that serum 25(OH)D_3_ levels decreased with increment of FM and VFA, and that the inverse influence of obesity on serum 25(OH)D_3_ levels might be mostly attributable to the effects of VFA, irrespective of BMI.

Several demographic factors have been recognized for their effects on serum 25(OH)D concentrations, including sex, age, race, and even the season during which blood sampling occurred [20]. In a previous study of a large Caucasian population, decreased serum 25(OH)D levels (<75 nmol/L) were found to exist in significantly more of the obese subjects than in the non-obese subjects [21]. Another study of various ethnicities demonstrated inverse correlations of serum 25(OH)D_3_ levels with body weight, BMI, and WC [22]. In the current study, the subjects were selected to help minimize the effects of potential confounding factors (as detailed above); for example, each subject had NGT and resided in a single geographic region, were of a single race, reported similar dietary status, provided blood samples in a single season (to help ensure similar daily sunshine duration and potential exposure). The results indicated that serum 25(OH)D_3_ levels decreased in the overweight/obese and were negatively correlated with WC; these findings are consistent with the previous studies.

Although BMI is an adequate estimator of whole body fat and is used commonly in clinical analysis, it cannot distinguish fat mass from lean blocks. The accurate variables (FM and fat%) can help to compensate for the deficiency. Studies revealed that serum 25(OH)D levels were inversely correlated with fat% in adolescents and healthy women [23], [24]. The present study found that a decreasing trend for serum 25(OH)D_3_ levels were accompanied by increased levels of FM. Moreover, among the overweight/obese group in the present study, serum 25(OH)D_3_ levels were 14.17% lower for those individuals with a higher level of fat% compared to those with a lower level of fat%. These results indicate that whole body fat may exert an inverse impact on the levels of 25(OH)D_3_, to a certain extent.

Although WC can be considered as a brief anthropometric index of abdominal obesity, it cannot accurately reflect the content or location of the abdominal fat clearly or directly. As such, the International Diabetes Federation has recommended using computed tomography (CT) and MRI as the standard quantification methods for VFA and SFA [25]. Indeed, in a study of non-diabetic Caucasians, serum 25(OH)D levels were found to be independently associated with CT-determined SFA and VFA, but only the relationship with VFA remained significant after further stratified by BMI [26]. These results indicated that serum 25(OH)D concentrations were inversely correlated with visceral adiposity. In the present study, regional adipose deposits were measured in a precise manner using MRI and the results of analysis indicated that serum 25(OH)D_3_ levels decreased in conjunction with the increment of VFA. This finding is in accordance with the previous studies in diverse ethnicities [27], [28].

A particularly intriguing finding from the present study is that, regardless of overweight/obesity status (i.e. BMI category), individuals with higher VFA showed lower serum 25(OH)D_3_ levels and that only VFA and TG were identified as independent risk factors of serum 25(OH)D_3_ levels after adjustment for conventional confounders. Thus, it appears that, compared with total fat content, increased VFA may contribute more to decreased levels of serum 25(OH)D_3_.

Several putative mechanisms may explain the association observed between the decreased levels of serum 25(OH)D_3_ levels and the clinical measures of adiposity. For example, since vitamin D is fat-soluble, the increased adipose tissue that occurs in the obese state will expand the distribution of the pool of vitamin D, thereby reducing the overall concentration of serum vitamin D levels [29]. In turn, Vitamin D deficiency is also an important risk factor of obesity. Reduced levels of serum vitamin D can lead to a secondary elevation of parathyroid hormone (PTH), which may promote calcium influx into adipocytes and increase lipogenesis and reduce lipolysis [30], [31]. In addition, vitamin D is capable of inhibiting differentiation of preadipocytes via its suppression of peroxisome proliferator-activated receptor γ (PPARγ) expression and activation, thereby causing an increase in lipogenesis when serum vitamin D levels decrease [32].

Perturbed vitamin D status also appear to increase the risks of dyslipidemia. A previous study of Spanish subjects with BMI ≥40 kg/m^2^ showed that individuals with serum vitamin D deficiency had higher levels of TG [33]. Furthermore, a study of elderly Chinese demonstrated that serum 25(OH)D levels were inversely associated with TG in men, but not in women [34]. Consistent with these previous findings, the present study of adult Chinese men with NGT identified TG as an independent risk factor of serum 25(OH)D_3_ levels. The mechanism underlying this phenomenon may involve intracellular Ca^2+^; in this manner, an antilipolytic effect may be exerted, mainly by the activation of phosphodiesterase, leading to a decrease in cAMP and hormone-sensitive lipase phosphorylation [35].

Previous studies have also indicated that serum 25(OH)D levels are positively correlated with physical activity and negatively correlated with age [36]. However, this relationship was not observed in the current study population, possibly as a result of the relatively narrow age range which may have weakened the impact of age and physical exercise on 25(OH)D_3_.

Some limitations inherent to the current study’s design may have influenced the overall findings. First, the sample size was relatively small and the subjects recruited were mainly middle-aged and elderly, limiting the ability to generalize these findings to the more heterogeneous population. Second, the fact that PTH levels were not measured precluded the ability to determine whether or not the relationship between 25(OH)D_3_ and clinical measures of adiposity was caused by secondary hyperparathyroidism. Previous studies, however, have demonstrated that the relationship between serum 25(OH)D levels and obesity is independent of serum PTH levels [27]. In addition, individuals with abnormal serum calcium or taking calcium/vitamin D supplements were excluded from the study enrollment, which could have partially compensated for this deficiency. Finally, the cross-sectional design precluded the ability to identify the exact causal relationship between 25(OH)D_3_ and the clinical measures of adiposity.

## Conclusions

This study has provided the first evidence of adipose tissue, especially increased visceral adipose, being related to marked decreases in serum 25(OH)D_3_ levels in Chinese men with NGT.
